# Undernutrition increased the risk of loss to follow-up among adults living with HIV on ART in Northwest Ethiopia: a retrospective cohort study

**DOI:** 10.1038/s41598-022-27077-y

**Published:** 2022-12-29

**Authors:** Animut Alebel, David Sibbritt, Pammla Petrucka, Daniel Demant

**Affiliations:** 1 College of Health Science, Debre Markos University, Debre Markos, Ethiopia; 2 School of Public Health, Faculty of Health, University of Technology Sydney, Ultimo, NSW Australia; 3 College of Nursing, University of Saskatchewan, Saskatoon, Canada; 4 School of Life Sciences and Bioengineering, Nelson Mandela African Institute of Science and Technology, Arusha, Tanzania; 5 School of Public Health and Social Work, Faculty of Health, Queensland University of Technology, Kelvin Grove, QLD Australia

**Keywords:** Health care, Medical research, Risk factors

## Abstract

This study aims to examine the effect of undernutrition on loss to follow-up (LTFU) in adults living with human immunodeficiency virus (HIV) receiving antiretroviral therapy (ART) in Ethiopia. We conducted an institution-based retrospective cohort study using medical records of 844 adults living with HIV receiving ART between June 2014 and June 2020 at Debre Markos Comprehensive Specialized Hospital (Northwest Ethiopia). The effect of undernutrition on LTFU was examined using a proportional hazards regression model after adjusting potential confounders. The significance level was set at p < 0.05. At the end of the study period, 109 (12.9%) participants were considered LTFU, with an overall LTFU incidence of 5.3 per 100 person-years (95% CI 4.4, 6.4). The incidence of LTFU was higher in undernourished participants (8.2 per 100 person-years) compared to well-nourished participants (4.3 per 100 person-years). After adjusting for potential confounders, the adjusted risk of LTFU among undernourished participants was two times higher than in their well-nourished counterparts (AHR [adjusted hazard ratio]: 2.1, 95% CI 1.4, 3.2). This study found that undernutrition significantly increased the risk of LTFU among adults living with HIV on ART.

## Introduction

Although the human immunodeficiency virus (HIV) is a global public health concern, sub-Saharan Africa (SSA) is the most affected region. Of the 37.7 million people living with HIV (PLHIV) worldwide in 2020, more than two-thirds (67%) were from SSA^1^. Ethiopia is one of the SSA countries with a high prevalence of HIV^2^. Although there is no cure for HIV infection, antiretroviral therapy (ART), a combination of three or more antiretroviral drugs, enables PLHIV to live longer, healthier lives^3^. Since 2016, all PLHIV have been eligible to start ART as soon as possible, regardless of their clinical status^3^. As of December 2020, 25.7 million (73%) PLHIV were on ART worldwide^1^.

Despite the dramatic increase in access to ART, high rates of loss to follow-up (LTFU) remain a significant challenge, especially in resource-limited settings, with HIV-care related LTFU emerging as a public health issue in low- and middle-income countries (LMICs)^4,5^, including SSA^6^. A collaborative analysis has shown that 18.8% of adults living with HIV in SSA were lost to ART follow-up^7^. A systematic review and meta-analysis from Ethiopia found that LTFU among adults living with HIV was 15.2% throughout follow-up^8^. LTFU has been associated with various unfavourable treatment outcomes, such as frequent hospitalizations, high risk of treatment failure, high rate of mortality, and high risk of opportunistic infections (OIs)^9^. SSA based studies have shown that the most common risk factors of LTFU in adults living with HIV are poor ART adherence, being younger, being male, not disclosing HIV-status, advanced World Health Organization (WHO) clinical disease stage (III and IV), low CD4 cell count, not taking co-trimoxazole preventive therapy (CPT), not taking isoniazid preventive therapy (IPT), and being undernourished^10–14^.

Various studies conducted in Africa have shown that undernutrition is significantly associated with a higher risk of LTFU in adults living with HIV receiving ART^14–18^. Undernutrition may indirectly increase the risk of LTFU as it accelerates disease progression from HIV to acquired immunodeficiency syndrome (AIDS) and increases the occurrence and recurrence of OIs^19,20^. Undernourished patients may not return to the health facility for ART refill and miss their appointments due to frequently being sick or feeling generally unwell^21^. However, a Ugandan study showed that overweight (BMI > 30 kg/m^2^) participants are at lower risk of LTFU^22^. This could be explained as being overweight is the leading risk factor of non-communicable diseases (NCDs), especially hypertension and diabetes^23^. Overweight HIV-infected patients may benefit from the program, as regular health education on medication adherence is a component of care for patients with NCDs^24^.

The United Nations Sustainable Development Goal (SDG #3) aimed to end the epidemic of HIV/AIDS by 2030^25^. To meet this ambitious target, optimizing retention in ART care is an effective strategy. In line with this agenda, the Ethiopian government has adapted various strategies to reduce LTFU in PLHIV, such as adherence support through phone calls and tracing patients who have missed their appointments. Despite these interventions, LTFU from ART is still a significant challenge for health care professionals. As mentioned above, undernutrition is one of the main factors significantly associated with LTFU among PLHIV. Understanding the impact of undernutrition on LTFU is essential for designing and implementing evidence-based interventions. However, studies examining the impact of undernutrition on LTFU in this population are scarce in SSA, and none has been conducted in Ethiopia. Thus, this study aimed to examine the impact of undernutrition on LTFU in adults living with HIV receiving ART by considering undernutrition as an exposure variable. The findings of this study will help clinicians to develop targeted interventions to reduce undernutrition related LTFU. It will also inform further interventional studies.

## Methods

### Study design, area, and period

This study used a part of data came from a large retrospective cohort study conducted to examine the effects of undernutrition on treatment outcomes among adults living with HIV on ART between June 2014 and June 2020 at Debre Markos Comprehensive Specialized Hospital (DMCSH), Northwest Ethiopia. Therefore, the detailed methodology was described in our previously published two papers^26,27^. DMCSH is the only referral hospital in the East Gojjam Zone in the Amhara Region, located 300 km from Addis Ababa, the capital of Ethiopia, and 255 km from Bahir Dar, the capital of the Amhara region. It serves over 3.5 million people in East Gojjam Zone and neighbouring zones. The hospital commenced offering chronic HIV care and ART services in 2005. Out of the total 1209 PLHIV who started ART at DMCSH between June 2014 and June 2020, 1177 (97.4%) were adults. Adulthood was defined as patients’ ≥ 15 years of age since this population is considered and treated as adults in Ethiopia for treatment purposes.

### Study participants

The study population was all adults living with HIV who started ART at DMCSH between June 2014 and June 2020 and received ART for at least one month. All patients aged 15 years or older were defined as adults, consistent with the Ethiopian ART treatment guidelines^28^. Patients were excluded from the study if they were transferred to DMCSH without baseline information and without a recorded date for the outcome of interest (LTFU). Furthermore, pregnant women were excluded as the nutritional assessment is different from other HIV-positive adults^29^.

### Sample size and sampling procedures

**Figure 1:**
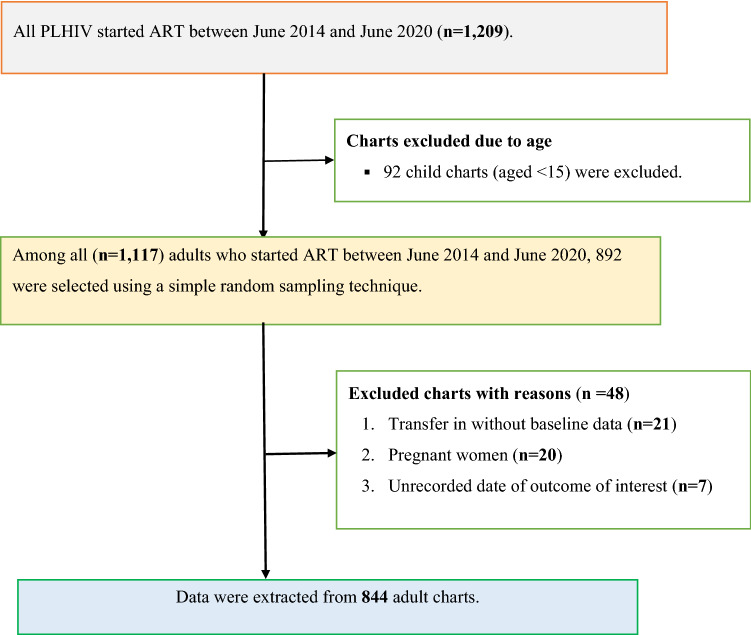
Study participants’ recruitment process at Debre Markos Comprehensive Specialized Hospital in Northwest Ethiopia, between June 2014 and June 2020.

The minimum required sample size was determined using an independent cohort study formula and calculated using Open Epi Version 3^30^. Undernourished participants were considered as the exposed group, and their well-nourished counterparts were considered as the unexposed group. The following parameters were taken into consideration: proportion of unexposed (well-nourished) group with lost to follow-up (P_0_ = 19%); proportion of exposed (undernourished) group with lost to follow-up (P_1_ = 27%); r of 1:1; α of 5%; power of 80%; and Z_α/2_ of 1.96. The P_0_ and P_1_ values were taken from a previous Ethiopian study^10^. The sample size calculated based on the above parameters was 802. Allowing for 10% contingency, the records of 892 study participants were selected from the total records of HIV-positive adults who started ART at DMCSH between June 2014 and June 2020 (n = 1177) using a computer-generated simple random sampling technique. Initially, a list containing the medical registration number (MRN) of all adults living with HIV initiated ART between June 2014 and June 2020 was obtained from the health management information system unit of the DMCSH. Then, a random number was generated for each patient using Microsoft™ Excel. Lastly, randomly generated numbers were used to select a sample of 892 participants from all adults living with HIV who started ART at DMCSH between June 2014 and June 2020. The final sample included 844 records after 48 records were excluded (see Fig. 1).

### Data collection and quality control

Data from the medical records of HIV patients were extracted using a standardized data extraction checklist developed using the current Ethiopian ART treatment guidelines. The data extraction checklist included sociodemographic characteristics, clinical and immunological characteristics, and follow-up characteristics. Variables included in the sociodemographic characteristics were age, sex, marital status, level of education, occupation, residence, HIV-status disclosure, and family size. Clinical and immunological variables included baseline OIs, nutritional status, functional status, CD4 cell counts, WHO clinical staging, and haemoglobin (Hgb) level. Variables extracted from the follow-up data included ART eligibility criteria, ART regimen, OIs during follow-up, ART adherence, regimen change, CPT, IPT, treatment failure, and patient outcomes. Sociodemographic measurements recorded at ART initiation were considered as baseline data. However, for other variables, such as laboratory tests, the most recent values were used as predictors. Two epidemiologists specialized in HIV currently working at DMCSH were recruited as data collectors.

### Outcome of the study

The outcome of this study was the occurrence of loss to follow-up (LTFU) after ART initiation. LTFU was defined as patients missing an ART appointment for at least one month^28^. At the end of follow-up, participants were classified as event (lost) or censored (not lost). Censored was considered when participants died or were still alive under ART at the end of follow-up (30th of June 2020) or formally transferred to other health facilities. The follow-up time was calculated in months from the date of ART initiation until the date of event (LTFU) or censoring (other than lost).

### Expose variable

Undernutrition was diagnosed when participants had a body mass index (BMI) below 18.5 kg/m^2^^31^. All participants (n = 227) with a BMI of below 18.5 kg/m^2^ were the exposure group of this cohort.

### Covariates and operational definitions

Covariates (confounders) included sociodemographic variables, clinical and immunological variables, and follow-up variables, as mentioned in the data collection section.

Residency was classified as urban and rural. Under the Ethiopian Central Statistical Agency (CSA), urban is considered if the local area contains 2000 or more inhabitants. In addition, urban areas include all administrative capitals of regions, zones, and woredas (districts), with at least 1000 people primarily engaged in non-agricultural activities or areas that the administrative official declares to be urban^32^. Family size refers to the number of individuals in the family. Family size was classified as < three or ≥ three standards established by previous research^33^.

Disclosureof HIV status was recorded as disclosed and not disclosed. According to current Ethiopian ART guidelines, patients are considered as disclosed their HIV status if they have disclosed their HIV status to at least one person (i.e., sexual partner or family member)^28^. ART adherence was classified as good, fair, or poor, calculated from the total monthly dose of ART drugs (n = 60). Good is compliance equal to or greater than 95% or ≤ 3 missed doses per month; fair is defined as 85–94% compliance or between 4 and 8 missing doses per month; and poor as compliance of less than 85% or ≥ 9 missed doses per month^28^.

Functional status was classified as working, ambulatory, and bedridden. Working was defined as being capable of going out of home and do routine activities, including daily work. Ambulatory was defined as capable of self-care and being able to use the toilet unsupported. Bedridden was defined as incapable of basic self-care (i.e., not able to use toilet without support)^28^.

The WHO staging of HIV has four levels to determine the degree of immunodeficiency based on CD4 cell count: no significant immunosuppression (CD4 > 500 cells/mm^3^), mild immunosuppression (CD4: 350–499 cells/mm^3^), advanced immunosuppression (CD4: 200–349 cells/mm^3^), and severe immunosuppression (CD4 < 200 cells/mm^3^)^34^. In addition, the WHO has implemented a clinical staging system based on clinical symptoms beyond CD4 cell count listed herein^28^:

- *Clinical stage I* Usually asymptomatic, except persistent generalized lymphadenopathy.
- *Clinical stage II* One of the following clinical presentations is present: moderate unexplained weight loss (5–10% of presumed or measured body weight), recurrent upper respiratory tract infections (sinusitis, tonsillitis, otitis media, and pharyngitis), herpes zoster, angular cheilitis, recurrent oral ulceration, papular pruritic eruption, fungal nail infections, and seborrheic dermatitis.
- *Clinical stage III* At least one of the following conditions is present: unexplained severe weight loss (> 10% of presumed or measured body weight), unexplained chronic diarrhoea > one month, unexplained persistent fever (intermittent or constant > one month), persistent oral candidiasis, oral hairy leucoplakia, pulmonary tuberculosis, severe bacterial infections (such as pneumonia, empyema, pyomyositis, bone or joint infection, meningitis, bacteraemia), acute necrotizing ulcerative stomatitis, gingivitis or periodontitis, unexplained anaemia, neutropoenia, and/or chronic thrombocytopaenia.
- *Clinical stage IV* At least one of the following severe conditions is present: HIV wasting syndrome, pneumocystis jirovecii, recurrent severe bacterial pneumonia, chronic herpes simplex infection (orolabial, genital or anorectal of more than one month’s duration or visceral at any site), oesophageal candidiasis (or candidiasis of trachea, bronchi or lungs), extra pulmonary tuberculosis, Kaposi sarcoma, cytomegalovirus infection (retinitis or infection of other organs), central nervous system toxoplasmosis, HIV encephalopathy, extra pulmonary cryptococcosis, disseminated non-tuberculous mycobacterial infection, progressive multifocal leukoencephalopathy, chronic cryptosporidiosis, chronic isosporiasis, disseminated mycosis (extra-pulmonary histoplasmosis, coccidioidomycosis), lymphoma (cerebral or B-cell non-Hodgkin), symptomatic HIV-associated nephropathy or cardiomyopathy, recurrent septicaemia (including non-typhoidal salmonella), invasive cervical carcinoma, and atypical disseminated leishmaniasis.

### Missing data handling

Data for 208 (24.6%) CD4 cell counts and 50 (5.9%) haemoglobin (Hgb) levels were not available from patient records. Multiple imputation (MI) was undertaken using a multivariate normal imputation model. Before MI, Little’s MCAR test^35^ was performed to check if the values are missing at random (MCAR). Moreover, we checked the patterns and mechanisms of missing values. Variables included in the imputation model were sex, residence, WHO clinical disease staging, ART adherence, nutritional status, baseline OIs, CPT, and IPT. Finally, diagnostic plots for multiple imputation were employed to assess the distributions of observed, imputed, and completed data (multiple imputation diagnostic test).

### Statistical analysis

Chi-square (χ^2^) tests were used to compare the frequencies of sociodemographic, clinical, and follow-up characteristics between undernourished and well-nourished groups. The Kaplan–Meier survival curve was used to visualize the survival time of loss to follow-up. A generalized log-rank test was used to compare survival curves between undernourished (exposed) and well-nourished (non-exposed) groups. Bi-variable and multivariable proportional hazards regression models were fitted. The proportionality assumption of the proportional hazards regression model was assessed using the Schoenfeld residual test. In the bi-variable analysis, variables with p-values ≤ 0.25 were included in the multivariable analysis for adjusting confiders. Results from the final model were reported as adjusted hazard ratios (AHRs) with 95% confidence intervals (CIs). The significance level was set at p < 0.05. All statistical analyses were carried out using Stata™ Version 16.

### Ethics approval and consent to participate

Ethical approvals and permissions were granted from the DMCSH Medical Director’s Office, the University of Technology Sydney Medical Research Ethics Committee (ETH20-5044), and the Amhara Regional Public Health Research Ethics Review Committee (Ref. no: 816). All methods were performed in accordance with the relevant guidelines and regulations. As the study was based on existing medical records of PLHIV, participants' verbal or written informed consent was not feasible, and a waiver of consent was granted. Data were entirely de-identifiable for the authors and data collectors, as the data abstraction tool did not include participants’ unique ART numbers and names.

## Results

### Participants’ sociodemographic characteristics

Of the 844 participants included in the final sample, more than three-quarters (78.4%; n = 662) were from urban areas. The cohort included 227 (26.9%) undernourished and 617 (73.1%) well-nourished participants. The median age of participants at ART initiation was 32 years (IQR: 26–40). More than half (59.0%; n = 498) were female, 18.3% (n = 154) had never married, and 25.8% (n = 218) had attended at least primary school. Homemakers accounted for 16.9% (n = 143) of the sample; 66.9% (n = 565) had disclosed their HIV-status, and 55.3% (n = 447) were from a family of less than three people (see Table 1).

**Table 1:** Socio-demographic characteristics of undernourished and well-nourished participants at Debre-Markos Comprehensive Specialized Hospital, Northwest Ethiopia (n = 844).

Variables	Undernourished n (%)	Well-nourished n (%)	Total n (%)	p-values
**Residence**				0.001**
Urban	161(70.9)	501 (81.2)	662 (78.4)	
Rural	66 (29.1)	116 (18.8)	182 (21.6)	
**Age (years of age)**				0.679
15–24	51 (22.5)	135 (21.9)	186 (22.0)	
25–34	73 (32.2)	208 (33.7)	281 (33.3)	
35–44	69 (30.4)	200 (32.4)	269 (31.9)	
≥ 45	34 (15.0)	74 (12.0)	108 (12.8)	
**Sex**				0.018**
Male	108 (47.6)	238 (38.6)	346 (41.0)	
Female	119 (52.4)	379 (61.4)	498 (59.0)	
**Marital status**				0.002**
Single	56 (24.7)	98 (15.9)	154 (18.3)	
Married	88 (38.8)	303 (49.1)	391 (46.3)	
Divorced	53 (23.4)	163 (26.4)	216 (25.6)	
Widowed	30 (13.2)	53 (8.6)	83 (9.8)	
**Level of education**				0.665
No formal education	70 (30.8)	186 (30.2)	256 (30.3)	
Primary	56 (24.7)	162 (26.3)	218 (25.8)	
Secondary	66 (29.1)	158 (25.6)	224 (26.6)	
Tertiary	35 (15.4)	111 (18.0)	146 (17.3)	
**Occupation**				0.003**
Daily labourer	37 (16.3)	102 (16.5)	139 (16.5)	
Merchant	37 (16.3)	130 (21.1)	167 (19.8)	
Farmer	48 (21.2)	70 (11.4)	118 (14.0)	
Employed	41 (18.1)	142 (23.0)	183 (21.7)	
Student	18 (7.9)	29 (4.7)	47 (5.6)	
Homemaker	32 (14.1)	111 (18.0)	143 (16.9)	
Others	14 (6.2)	33 (5.4)	47 (5.6)	
**HIV-status disclosure**				
Disclosed	155 (68.3)	410 (66.5)	565 (66.9)	0.616
Not disclosed	72 (31.7)	207 (33.5)	279 (33.1)	
**Individuals/household**				
< 3 individuals	125 (55.1)	342 (55.4)	447 (55.3)	0.925
≥ 3 individuals	102 (44.9)	275 (44.6)	377 (44.7)	

### Clinical, immunological, and medication-related characteristics

Forty percent (n = 337) of patients had at least one opportunistic infection at ART initiation, and most (83.3%; n = 703) participants were classified as working functional status. Nearly a third (31.3%; n = 264) had severe immunodeficiency, and 43.7% (n = 369) were in WHO clinical stage I. The majority (80.2%; n = 677) of patients were not anaemic at ART initiation. More than half (55.5%; n = 468) started ART through a test and treat approach. Most participants (89.8%; n = 758) started an Efavirenz-based ART regimen, and 31.4% (n = 264) had a regimen change from baseline. CPT and IPT was not received by 26.9% (n = 227) and 37.6% (n = 317) of participants, respectively. During the study period, 23 (2.7%) patients experienced ART treatment failure, and 267 (31.6%) patients developed new OIs (Table 2).

**Table 2:** Clinical, immunological, and medication-related characteristics of undernourished and well-nourished participants at Debre-Markos Comprehensive Specialized Hospital, Northwest Ethiopia (n = 844).

Variables	Undernourished n (%)	Well-nourished n (%)	Total n (%)	p-value
**Baseline OIs**				< 0.001**
Yes	114 (50.2)	223 (36.1)	337 (39.9)	
No	113 (49.8)	394 (63.9)	507 (60.1)	
**Functional status**				< 0.001**
Working	160 (70.5)	543 (88.0)	703 (83.3)	
Ambulatory/ bedridden	67 (29.5)	74 (12.0)	141 (16.7)	
**Immunodeficiency**				< 0.001**
Not significant	95 (41.9)	169 (27.4)	224 (26.5)	
Mild	53 (23.4)	137 (22.2)	166 (19.7)	
Advanced	44 (19.4)	122 (19.8)	190 (22.5)	
Severe	35 (15.4)	189 (30.6)	264 (31.3)	
**WHO clinical staging**				< 0.001**
Stage I	74 (32.6)	295 (47.8)	369 (43.7)	
Stage II	60 (26.4)	180 (29.2)	240 (28.4)	
Stage III	74 (32.6)	114 (18.5)	188 (22.3)	
Stage IV	19 (8.4)	28 (4.5)	47 (5.6)	
**Haemoglobin level**				0.001**
Anaemic	62 (27.3)	105 (17.0)	167 (19.8)	
Not anaemic	165 (72.7)	512 (83.0)	677 (80.2)	
**ART eligibility criteria**				0.174
Immunological	82 (36.1)	203 (32.9)	285 (33.8)	
Clinical	30 (13.2)	61 (9.9)	91 (10.8)	
Test and treat	115 (50.7)	353 (57.2)	468 (55.5)	
**ART regimens**				0.442
Efavirenz-based	199 (87.7)	559 (90.6)	758 (89.8)	
Nevirapine-based	7 (3.1)	16 (2.6)	23 (2.7)	
Dolutegravir-based	21 (9.3)	42 (6.8)	63 (7.5)	
**ART adherence**				0.015**
Good	157 (69.2)	477 (77.3)	634 (75.1)	
Fair/ poor	70 (30.8)	140 (22.7)	210 (24.9)	
**ART regimen change**				0.378
Yes	66 (20.1)	199 (32.3)	265 (31.4)	
No	161 (70.9)	418 (67.8)	579 (68.6)	
**Taking IPT**				0.007**
Yes	125 (55.1)	402 (65.2)	527 (62.4)	
No	102 (44.9)	215 (34.9)	317 (37.6)	
**Taking CPT**				0.022**
Yes	179 (78.9)	438 (71.0)	617 (73.1)	
No	48 (21.2)	179 (29.0)	227 (26.9)	
**ART failure**				0.297
Yes	4 (1.8)	19 (3.1)	23 (2.7)	
No	223 (98.2)	589 (96.9)	821 (97.3)	
**OIs during follow-up**				0.004**
Yes	89 (39.2)	178 (28.8)	267 (31.6)	
No	138 (60.8)	439 (71.2)	577 (68.4)	

### Incidence of loss to follow-up

**Figure 2:**
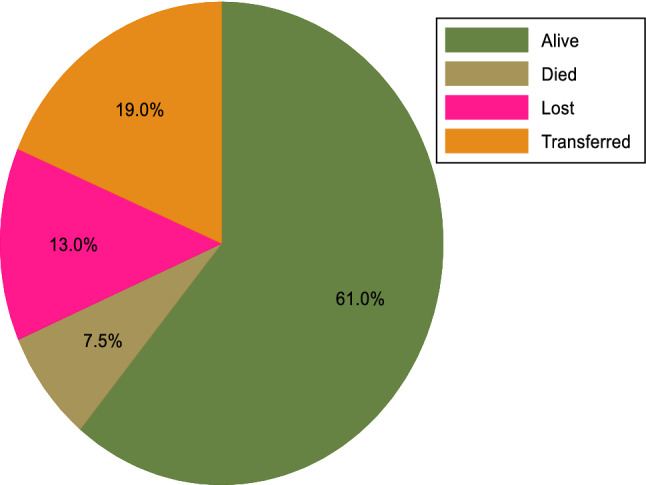
Treatment outcomes of adults living with HIV on ART at Debre Markos Comprehensive Specialized Hospital, Northwest Ethiopia, 2021.

**Figure 3:**
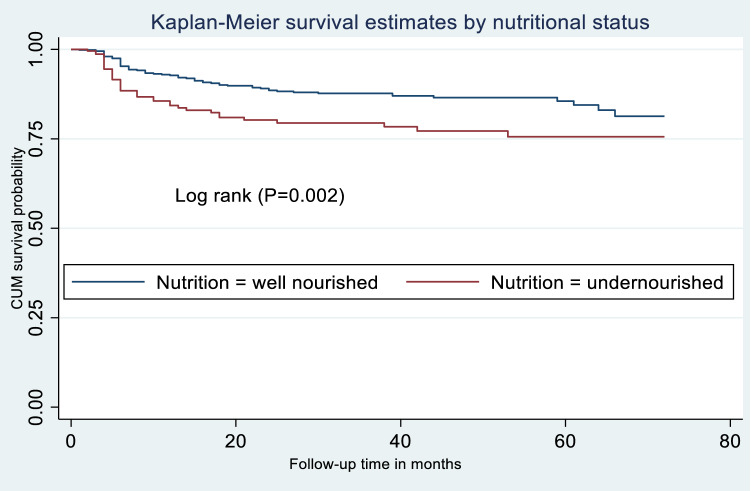
Kaplan–Meier survival curves to compare LTFU among undernourished and well-nourished adults living with HIV on ART at Debre Markos Comprehensive Specialized Hospital, Northwest Ethiopia, 2021.

The minimum follow-up time was one month with a maximum of 72 months, and a total follow-up time of 24,850 person-months. At the end of follow-up, 7.5% (n = 63) of participants had died, 12.9% (n = 109) were lost to follow-up, 18.6% (n = 157) were transferred out to other health facilities, and 61.0% (n = 515) were alive and on ART (see Fig. 2). Of all 63 deaths, 26 (42.3%) were from the undernourished group. Among the total lost cases, 84 (77.1%) were lost in the first year of follow-up. The overall incidence rate of LTFU in all participants was 5.3 per 100 person-years (95% CI 4.4, 6.4). However, a higher rate of LTFU (LTFU = 8.2; 95% CI 6.0, 11.1) was recorded in undernourished participants than well-nourished participants (LTFU = 4.3, 95% CI 3.4; 5.5). As shown in Fig. 3, the mean survival time of undernourished participants was significantly shorter than the mean survival time of well-nourished participants (p = 0.002).

### Effect of undernutrition on loss to follow-up

We performed a bi-variable analysis and variables with p-values ≤ 0.25 were included in the multivariable analysis to adjust for potential confounding variables. Variables included in the final model were residence, sex, functional status, baseline OIs, nutritional status, CD4 cell counts, WHO clinical stating, ART eligibility criteria, ART regimens, taking CPT, and taking IPT. After adjusting for potential confounders, the adjusted risk of LTFU among undernourished participants was two times higher than their well-nourished counterparts (AHR: 2.1, 95% CI 1.4, 3.2) (Table 3).

**Table 3:** The effect of undernutrition on LTFU among adults living with HIV on ART at Debre Markos Comprehensive Specialized Hospital, Northwest Ethiopia, 2021.

Variable	Loss to follow-up		CHR (95% CI)	AHR (95% CI)	P-value
	Event	Censured			
**Nutritional status**					
Undernourished	41	186	1.8 (1.2, 2.7)	2.1(1.4, 3.2)	< 0.001
Well-nourished	68	549	Ref	–	–

## Discussion

This retrospective cohort study examined the effects of undernutrition on LTFU among adults living with HIV initiated ART between 2014 and 2020 at DMCSH, Northwest Ethiopia. The study found that the rate of LTFU (8.2 per 100 person-years) in undernourished participants was higher compared to well-nourished participants (4.3 per 100 person-years); with the risk of LTFU in undernourished patients, being double that of well-nourished patients.

The overall incidence of LTFU in our study (5.3 per 100 person-years) is consistent with a study conducted in Zimbabwe (5.75 per 100 person-years)^36^. However, our finding is lower than studies conducted in Uganda (7.5 per 100 person-years)^22^ and Ethiopia (10.9 per 100 person-years)^10^. The above variations might be due to differences in sample sizes, follow-up periods, study settings, and population characteristics. In this regard, the follow-up periods of Ugandan^22^ and previous Ethiopian studies^10^ were three and four years, respectively. The follow-up period of our study was six years. In addition, our study included a referral hospital, whereas the Ugandan study included both hospital and health centres. Therefore, the lower LTFU rate in this study might be due to the differences in the quality of care provided at hospitals and health centres, as hospitals provide advanced care and commonly have more specialised staff.

The study found that of the total lost cases, more than three-fourths (n = 84, 77.1%) were seen in the first year of follow-up. This finding is supported by a study conducted in Guinea, which reported LTFU at one year was 42%^17^. A study from South Africa also revealed that half of the lost cases were observed in the first six months of ART follow-up^37^. A Nigerian study also found that half (51%) of the LTFU cases occurred within the first 30 days following ART initiation. The high rate of LTFU in the early stage of ART follow-up might be because newly diagnosed individuals visiting ART clinics may not be fully prepared to engage in HIV care, as ART is a lifelong medicine, requiring repeated counselling, and psychological preparation^28^. Although rapid ART initiation has improved the clinical outcomes of patients, particularly for those with very low CD4 cell counts, observational studies have shown that starting ART on the same day as HIV diagnosis may increase the risk of LTFU because patients need frequent counselling and psychological preparation before starting ART^38^. The WHO guidelines also mentioned the limitation of the test and treat approach, such as it increases the risk of LTFU^39^. Another possible explanation of high LTFU in the first year of follow-up could be due to ART-associated adverse drug reactions that are very common during the first year of ART. Studies suggested that ART-related adverse reactions negatively affect adherence, indirectly affecting LTFU^40–42^.

Our study found that the risk of LTFU in undernourished patients was two times higher as compared to their well-nourished counterparts. This finding is in line with studies conducted in Tanzania^14^, Uganda^22^, South Africa^43^, and Malawi^16^. This may be the results of undernourished patients being more likely to have underlying health conditions and eat less nutritious foods, which may directly impair clinic attendance^21^. In addition, food insecurity and undernutrition are significant predictors of poor ART adherence^44,45^. A qualitative study from Ugandan identified different mechanisms that explain how food insecurity leads to poor ART adherence^46^. Accordingly, patients believe that in the absence of food, ART increases appetite and causes hunger. Patients also believe that the side effects of ART can be aggravated without access to adequate food. Another reason is that they experienced competing demands of paying for food costs, transportation costs, and medical expenses. Lastly, they forget or are unable to take ART while working or searching for food^46^.

Undernutrition could also indirectly contribute to a higher risk of LTFU by increasing disease progression from HIV to AIDS stage in PLHIV^47^. Our study also showed that out of all the registered deaths (n = 63), 26 cases (42.3%) were from undernourished individuals. A systematic review and meta-analysis from LMICs found that advanced WHO clinical disease staging was significantly associated with higher risk of LTFU^48^. Advanced WHO clinical disease stage increases the risk of LTFU in various ways. Patients with WHO stage III or IV are more likely to have OIs and are bedridden most of the time, which can make it difficult for them to stay engaged in HIV care. In addition, HIV patients with advanced disease stage are at higher risk of premature death from severe OIs, especially during the first year. Therefore, these patients may have died at home but were not reported due to a passive surveillance system.

### Strengths and limitations of the study

One strength of our study is that it included relatively large sample size (n = 844). As we used a cohort study design, we were able to calculate the incidence rate of LTFU. However, this study has some limitations. Some important confounding factors, such as distance from the healthcare facilities and viral load, were unavailable due to data incompleteness. Moreover, the actual incidence of LTFU might be underestimated due to incomplete recording. Conversely, the actual incidence of LTFU may be overestimated because some patients classified as LTFU may have died or have started ART in other health care facilities without sufficient recording.

## Conclusion

Although Ethiopia has achieved remarkable success in increasing access to ART, LTFU is still a significant concern. The study found that undernutrition significantly increased the risk of LTFU among adults living with HIV on ART. This finding implies that different stakeholders should undertake various nutritional interventions and practical approaches to reduce LTFU and improve treatment outcomes in adults living with HIV. In addition, efforts must be made to improve the food security of people living with HIV across the country. Finally, further prospective studies by incorporating some confounders such as viral load and distance from health facilities are needed to examine the effects of undernutrition on LTFU.

## Data Availability

The data sets used and/or analysed for this study are available from the corresponding author on reasonable request.
